# Loss of calsyntenin paralogs disrupts interneuron stability and mouse behavior

**DOI:** 10.1186/s13041-022-00909-8

**Published:** 2022-03-12

**Authors:** Keita Mori, Michinori Koebis, Kazuki Nakao, Shizuka Kobayashi, Yuji Kiyama, Masahiko Watanabe, Toshiya Manabe, Yuichi Iino, Atsu Aiba

**Affiliations:** 1 Department of Biological Sciences, Graduate School of Science, The University of Tokyo, Tokyo, 113-0033 Japan; 2 Laboratory of Animal Resources, Center for Disease Biology and Integrative Medicine, Graduate School of Medicine, The University of Tokyo, 7-3-1 Hongo, Bunkyo-ku, Tokyo, 113-0033 Japan; 3 The Institute of Experimental Animal Sciences, Faculty of Medicine, Osaka University, Osaka, Japan; 4 Division of Neuronal Network, Department of Basic Medical Sciences, Institute of Medical Science, University of Tokyo, 4-6-1 Shirokanedai, Minato-ku, Tokyo, 108-8639 Japan; 5 Department of Anatomy, School of Medicine, Hokkaido University, Sapporo, Japan

**Keywords:** Calsyntenin, Mutant mice, CRISPR/Cas9, Triple knockout mice, Schizophrenia, Stress, Behavior

## Abstract

Calsyntenins (CLSTNs) are important synaptic molecules whose molecular functions are not fully understood. Although mutations in *calsyntenin* (*CLSTN*) genes have been associated with psychiatric disorders in humans, their function is still unclear. One of the reasons why the function of CLSTNs in the nervous system has not been clarified is the functional redundancy among the three paralogs. Therefore, to investigate the functions of mammalian CLSTNs, we generated triple knockout (TKO) mice lacking all CLSTN paralogs and examined their behavior. The mutant mice tended to freeze in novel environments and exhibited hypersensitivity to stress. Consistent with this, glucose levels under stress were significantly higher in the mutant mice than in the wild-type controls. In particular, phenotypes such as decreased motivation, which had not been reported in single *Clstn* KO mice, were newly discovered. The TKO mice generated in this study represent an important mouse model for clarifying the function of CLSTN in the future.

**Supplementary Information:**

The online version contains supplementary material available at 10.1186/s13041-022-00909-8.

## Introduction

Synaptic molecules play important roles in brain function, and disruption of these molecules leads to various types of psychiatric disorders. Although many molecules are involved in synaptic functions, there still remain a considerable number of molecules whose functions are to be revealed. CLSTNs are type I transmembrane proteins whose functions are not fully understood. CLSTNs contain two cadherin domains, laminin-alpha, neurexin, and sex hormone-binding globulin domains. CLSTNs are evolutionarily conserved across animal species and are expressed in the nervous system. Single orthologs, CASY-1 and Cals, have been identified in *Caenorhabditis elegans* [1] and *Drosophila melanogaster*, respectively, whereas three members are encoded in the mammalian genome. Importantly, *Clstn*s are widely expressed in the mammalian central nervous system (CNS) [2, 3]. Although the expression patterns of *Clstn*s in the CNS partially overlap, there are differences in the trends of each gene [3]. For example, in the hippocampus, *Clstn1* is strongly expressed in CA1 and CA3 pyramidal neurons and DG granule cells, whereas *Clstn2* and *Clstn3* are strongly expressed in CA2 and CA3 pyramidal neurons and interneurons and weakly expressed in CA1 pyramidal neurons [3].

CLSTNs in the brain are thought to be associated mainly with synapse adhesion and intracellular transport. In fact, simultaneous knockdown of three CLSTNs in the primary culture of hippocampal neurons reportedly reduced synaptic numbers [4]. CLSTN3, which is localized to postsynaptic sites, is expected to play an important role in synapse formation by directly interacting with neurexins on the presynaptic surface [5, 6].

In addition to their roles in synapse formation, CLSTNs also play a role in intracellular transport. All CLSTNs have a kinesin-binding motif (L/M-E/D-W-D-D-D-S), which binds directly to the kinesin-1 light chain and plays a role in intracellular vesicle transport [7–10]. Several important proteins have been reported as carriers of CLSTN1-positive cargo including amyloid precursor protein (APP) via binding to X11/Mint [11–13], N-methyl-D-aspartate receptors (NMDARs) [14], and γ-aminobutyric acid receptors type B (GABA_B_Rs) [13]. Although the functions of CLSTNs in intracellular trafficking have been reported for CLSTN1, the detailed mechanism(s) and involvement of CLSTN2 and CLSTN3 has not been clarified.

Several genetic studies have reported that CLSTNs are related to cognitive function in humans [15]. For example, association between *CLSTN2* alleles and significant differences in delayed word call was identified in a genome-wide association study in humans. Furthermore, Lipina et al*.* [16] have reported that *Clstn2* knockout (KO) mice display cognitive deficits. Interestingly, CASY-1, a homolog of *Clstns* in *C. elegans*, is also known to play an important role in several forms of learning [1, 17].

Although previous studies have focused on each of the three CLSTNs, CLSTNs are considered to have functional redundancy among the paralogs [4]. This suggests that there may be functions that have not been revealed due to functional redundancy because conventional studies have only used KO mice for each individual molecule. Therefore, to overcome the shortcomings of previous studies and to investigate the neurobiological role of CLSTNs in the mammalian brain, we generated *Clstn* triple KO (TKO) mice, in which all the paralogs of *Clstns* were mutated simultaneously. By performing behavioral and histological analyses in the generated TKO mice, we discovered novel phenotypes that suggest reduced motivation and increased stress responsiveness.

## Results

### Generation of *Clstn* triple knockout (TKO) mice

**Fig. 1:**
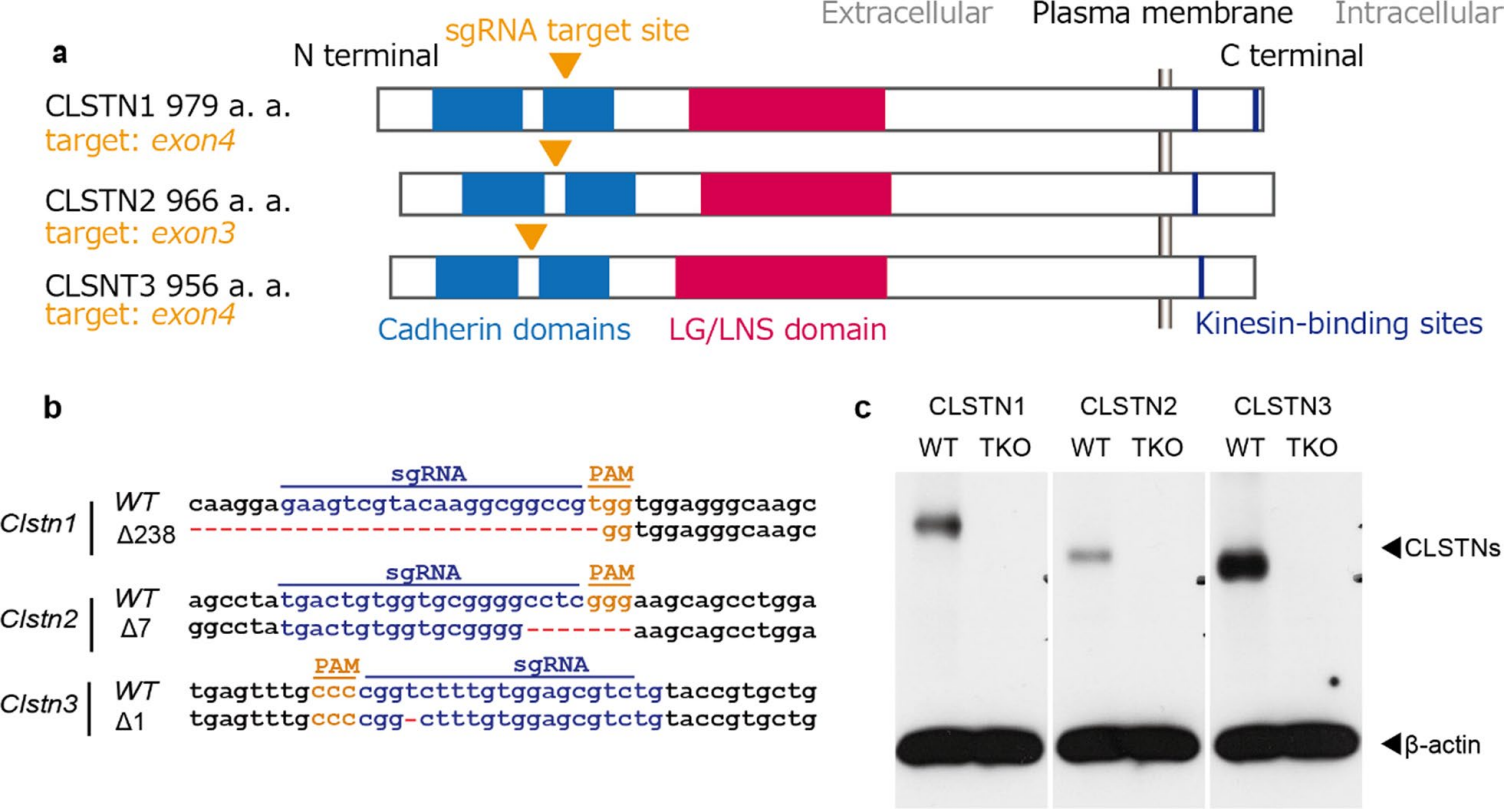
Generation of *calsyntenin (Clstn)* triple knockout (TKO) mice. **a** Representation of calsyntenin (CLSTN) domains and target regions of single-guide RNAs (sgRNAs). Each CLSTN has two cadherin domains (blue box), one LG/LNS domain (red box), and one or two kinesin-binding sites (blue line). The target region is designed to be upstream exon (exon 3 or exon 4) and is indicated by an arrowhead in the figure. **b** DNA sequences of modified *Clstn* genes in KO mice. The PAM sequences are highlighted in orange; the sgRNA-targeting sequences in blue; deletion mutations (−) in red. **c** Western blot of protein lysates of CLSTN-TKO whole brain shows the success of knockout at the protein level

**Fig. 2:**
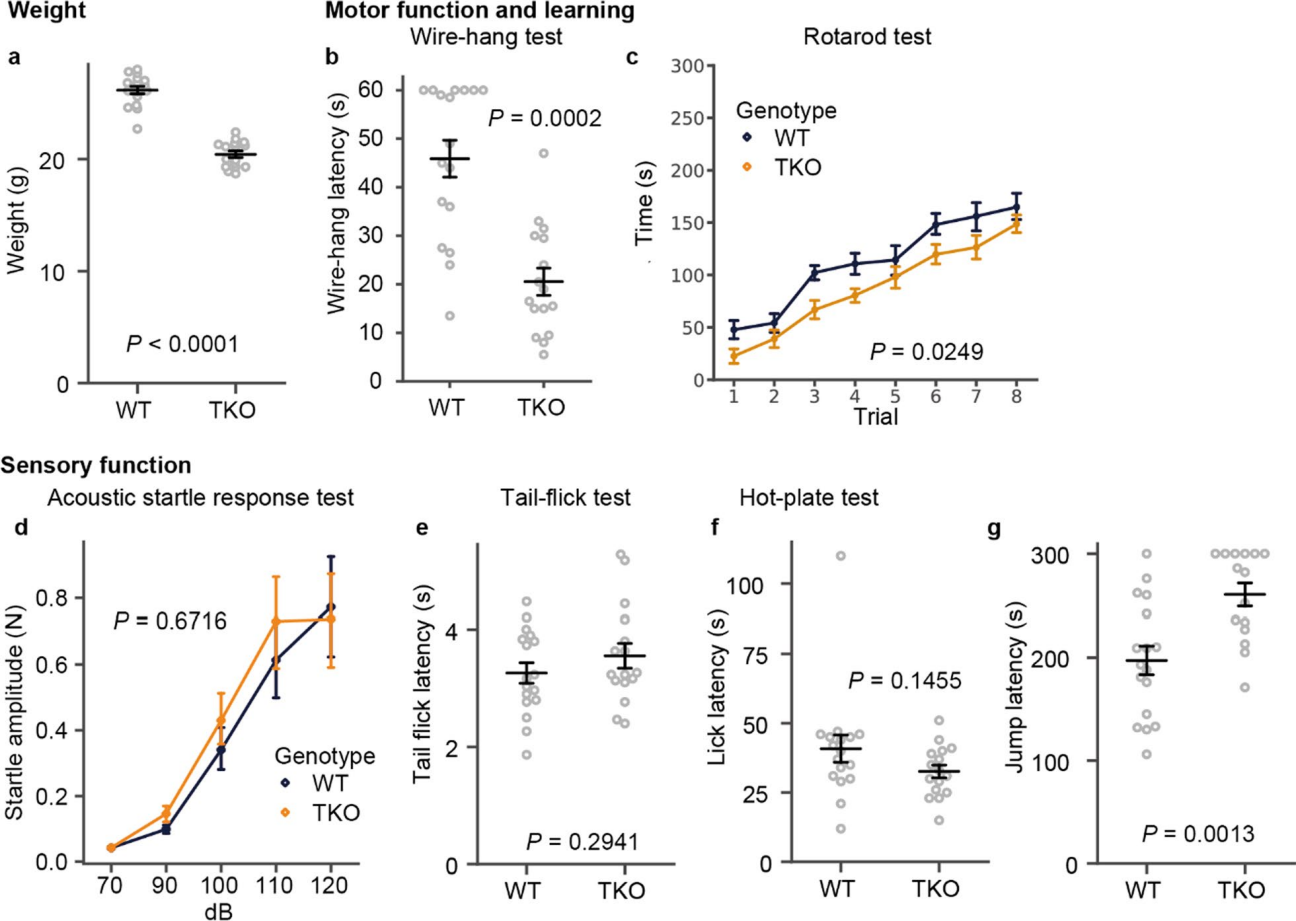
Sensory function and motor-learning test.** a** Weight of mice used in the behavioral battery test. **b**,** c** Wire-hang test score (**b**) and rotarod training result (**c**). **d–g** Acoustic startle (**d**) and pain sensation (**e–g**) for the sensory function test. All data are presented as the mean ± standard error of the mean (WT mice, n = 17; TKO mice, n = 16). *P* values for differences between genotypes were determined by Welch’s *t*-test (**a**, **e**, **f**, **g**), Wilcoxon rank-sum test (**b**), or calculation of genotype effect in two-way repeated-measures analysis of variance (**c**, **d**)

To systematically investigate CLSTN functions in vivo, we generated KO mice and tested their behavioral phenotypes. Given that functional redundancy between three *Clstn* paralogs was previously reported in experiments using cultured cells [4], we generated *Clstn* TKO mice, which carry mutations in all three paralogs of *Clstn*s using the CRISPR/Cas9 system. The upstream exon, which codes the N-terminal of the CLSTN protein, of each gene was targeted for deletion (Fig. 1a). DNA sequencing and Western blot analyses confirmed the KO of all *Clstn*s (Fig. 1b, c). The TKO was not embryonic lethal, and both male and female mice were fertile. In fact, the TKO mice rarely died after birth, safely reaching adulthood. However, the TKO mice tended to weigh less than wild-type (WT) mice in both sexes (Fig. 2a; Additional file 1: Fig. S1). Recent studies have revealed that CLSTN3 plays a role in body mass homeostasis in both hypothalamic neural circuits and adipocytes, and our results are consistent with those of previous studies [18, 19].

After confirming the general phenotypes described above, we performed behavioral, electrophysiological, and histological analyses of these TKO mice. In the following sections, we report the results of the behavioral and histological phenotyping.

### Behavioral tests reveal several new phenotypes related to stress response and motivation

To reveal the roles of CLSTNs in the CNS, we performed a behavioral battery test. *Clstn* TKO mice showed a few deficits in basic motor (Fig. 2b, c) and sensory (Fig. 2d–g) functions compared to WT mice. However, there was a significant difference in the weights of the mice used in the battery test (Fig. 2a). Although arm strength was significantly decreased in the TKO mice (Fig. 2b), motor learning skills were comparable to those of WT mice (Fig. 2c). The hearing of TKO mice was comparable to that of WT mice (Fig. 2d), and sensitivity to pain was normal (Fig. 2e, f) except for significantly longer latency to jump in the hot-plate test (Fig. 2g). As lick latency is comparable with WT mice (Fig. 2f), TKO mice clearly can sense the heat. The light weight (Fig. 2a) and decreased muscle strength (Fig. 2b) may partially explain the longer latency of jump response (Fig. 2g). From the series of experiments, we concluded that the sensory function of TKO mice is apparently normal.

### TKO mice showed hyperactivity in the familiar environment and took longer to start exploring in the novel environment

**Fig. 3:**
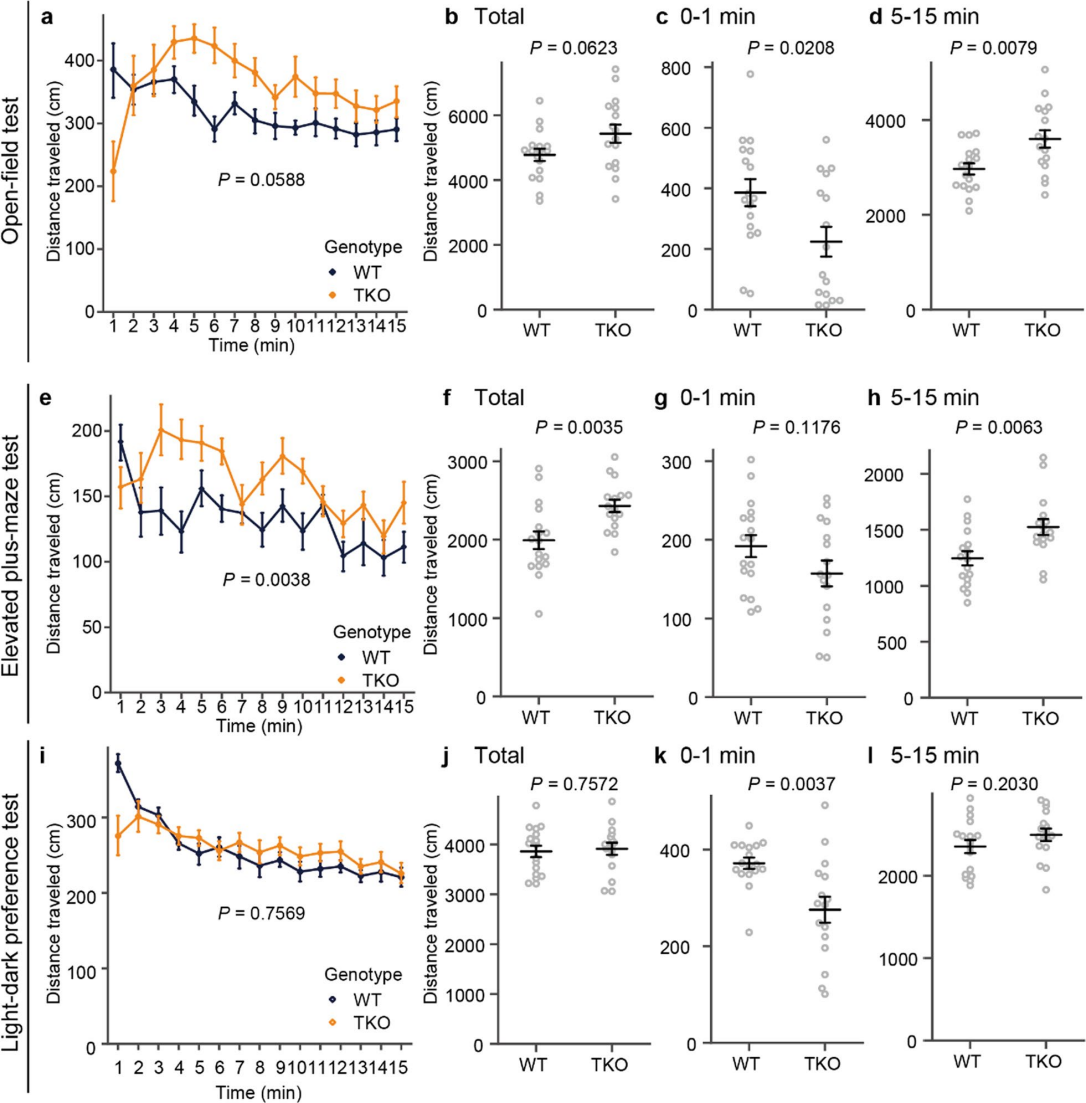
TKO mice showed hyperactivity in several behavioral tests. TKO mice showed hyperactivity in several behavioral tests indicated on the left end of the rows. **a–d** Distance traveled during the entire test (**a**, **b**), distance traveled during the first minutes (**c**), distance traveled during the last 10 min (**d**) for the open field test. **e–h** Distance traveled during the whole test (**e**, **f**), distance traveled during the first minutes (**g**), and distance traveled during the last 10 min (**h**) for the elevated plus-maze test. **i**–**l** Distance traveled during the whole test (**i**, **j**), distance traveled during the first minutes (**k**), and distance traveled during the last 10 min (**l**) for the light–dark preference test. All data are presented as the mean ± standard error of the mean (WT mice, n = 17; TKO mice, n = 16). *P* values for differences between genotypes were determined by Welch’s *t*-test (**b–d**, **f–h**, and **j–l**) or calculation of genotype effect in two-way repeated-measures analysis of variance (**a**, **e**, **i**)

Hyperactivity is one of the most common phenotypes in animal models of schizophrenia and bipolar disorder. We quantified the activity of TKO mice in the open-field test (Fig. 3a–d), elevated plus-maze test (Fig. 3e–h), and light–dark preference test (Fig. 3i–l). In the open-field test, elevated plus-maze test, and light–dark preference test, mice are first placed in novel environments. Usually, WT mice show more exploratory behavior in novel environments (at the first 1 min after the start) than in familiar conditions (5–15 min after the start), as actually observed in this study. In these tests, the trend of activity was different between novel and habituated environments (Fig. 3). The total distance traveled by TKO mice was significantly greater than that of control mice after habituated period of the open-field test (Fig. 3d) and elevated plus-maze test (Fig. 3h). Contrary to the hyperactive feature, for the first minute after being placed in a novel environment, TKO mice tended to freeze and be hypoactive compared with WT mice in the open field (Fig. 3c), elevated plus-maze (Fig. 3g), and light–dark preference tests (Fig. 3k).

### The level of anxiety of TKO mice was comparable to that of the wild-type mice

**Fig. 4:**
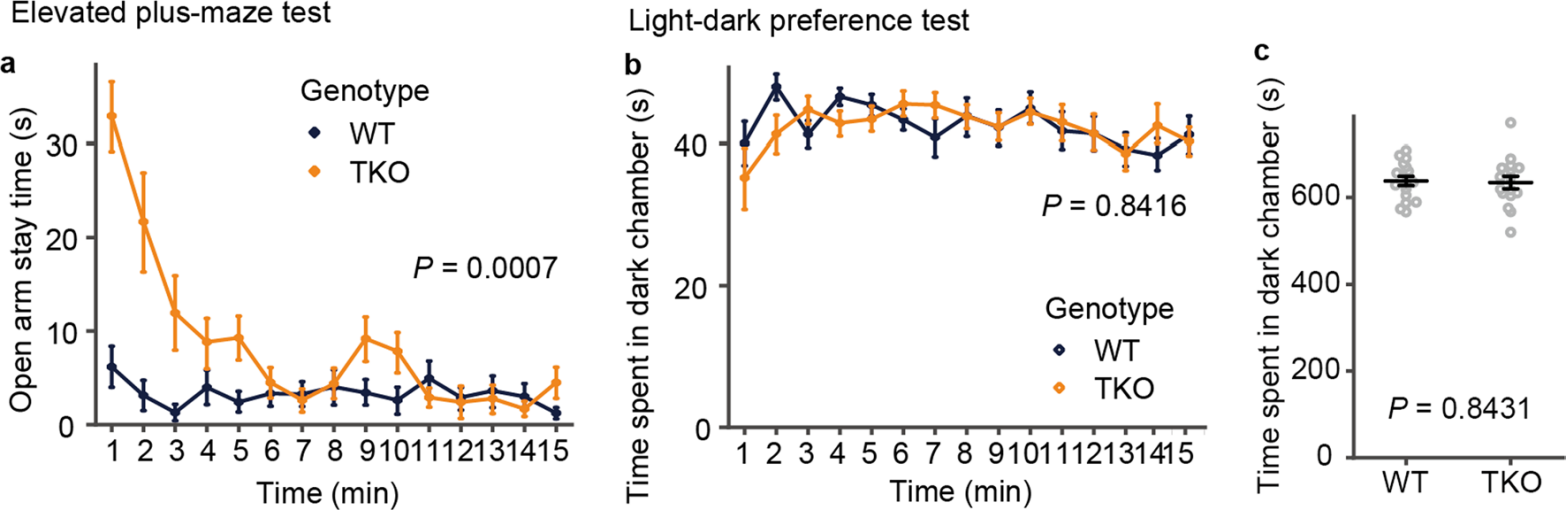
The tests for evaluating anxiety levels. Anxiety-related behavior of TKO mice. **a** Time spent in the open arms during the test. TKO mice showed a longer search time in the open arm immediately after the start of the test, but after 5 min, the time spent in the open arm was similar to that of the WT mice. **b**, **c** Time spent in the dark chamber for the light–dark preference test. In the light–dark preference test, WT and TKO mice showed similar levels of anxiety. All data are presented as the mean ± standard error of the mean (WT mice, n = 17; TKO mice, n = 16). *P* values for differences between genotypes were determined by Welch’s *t*-test (**c**) or calculation of genotype effect in two-way repeated-measures analysis of variance (**a**, **b**)

Next, we assessed anxiety-related behaviors using the elevated plus-maze and light–dark preference tests. Although TKO mice spent significantly more time in the open arms and tried to find the path to escape during the first 5 min, indicating that the anxiety level was decreased at the start, they later spent a similar amount of time as the WT mice (Fig. 4a). These results suggest that the level of motivation to escape from the high place was increased and the level of anxiety of TKO mice was reduced at the beginning, but after becoming familiar with the environment, the level of anxiety of TKO mice was comparable to those of the WT mice (Fig. 4a). In contrast, the light–dark preference test did not detect any difference in the time the mice spent in each chamber, which indicates that the anxiety level was comparable to that of the WT mice throughout the test (Fig. 4b, c).

### TKO mice tended to give up soon in the water-maze test

**Fig. 5:**
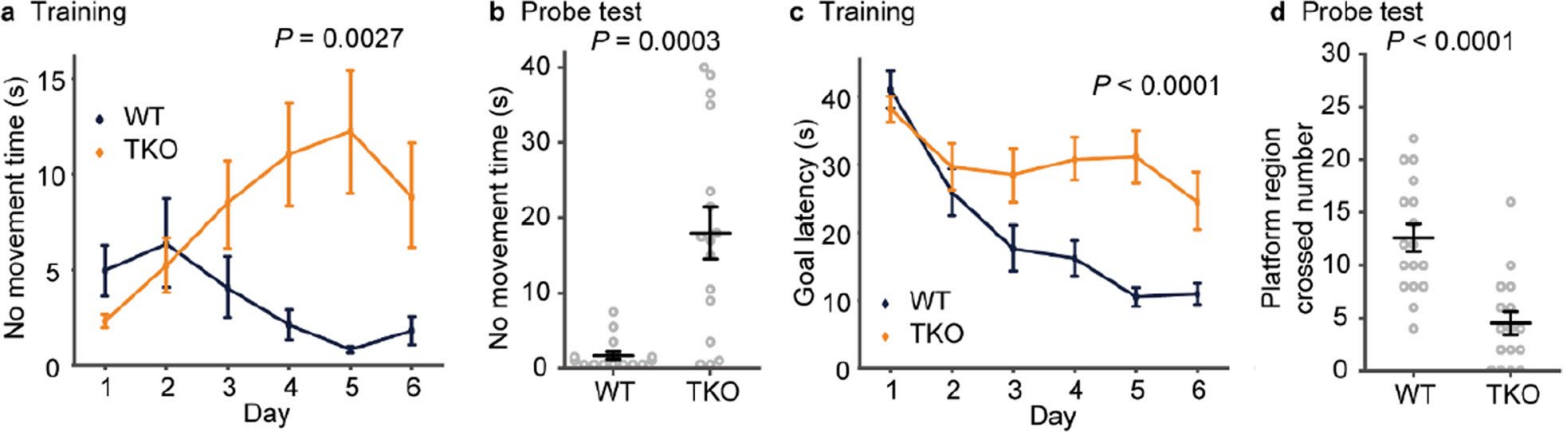
The water-maze test.** a–d** Morris water-maze test in TKO mice. In the Morris water-maze test, TKO mice gave up the search and showed increased time of inactivity after the second day (**a**). In the probe tests performed on the last day of the series of training, the TKO mice also showed lower motivation compared to WT mice (**b**). In TKO mice, the latency to platform arrival was longer than that in WT mice due to the increased time of inactivity and lower motivation (**c**). In probe tests performed on the last day of the training series, the TKO mice crossed the platform area less often (**d**). All data are presented as the mean ± standard error of the mean (WT mice, n = 17; TKO mice, n = 16). *P* values for differences between genotypes were determined by Welch’s *t*-test (**b**, **d**) or calculation of genotype effect in two-way repeated-measures analysis of variance (**a**, **c**)

To test the ability of hippocampus-dependent spatial memory, mice were subjected to the Morris water-maze task (Fig. 5). However, TKO mice stopped swimming on days 3–6 in the training session (Fig. 5a, b), which may reflect the abnormal stress responses of TKO mice. Since the TKO mice gave up swimming before they could complete the test in the probe test, they took longer to reach the platform (Fig. 5c), and the number of crossing the region in the pool where the target platform had been placed was reduced (Fig. 5d). This reduced motivation is a novel phenotype, which has not been reported in *Clstn* single KO mice [6, 16, 20]. Although we could not evaluate hippocampus-dependent learning abilities using Morris water maze, we assessed synaptic plasticity by examining long-term potentiation (LTP) induced at Schaffer collateral-CA1 synapses in the hippocampus of TKO mice and found that LTP of TKO mice was not distinguishable from that of WT mice (Additional file 1: Fig. S2).

### TKO mice showed a high freezing rate in the fear conditioning

In the fear conditioning test, another memory test, TKO mice showed a higher freezing rate in the chamber prior to fear conditioning (Additional file 1: Fig. S3a, b). After conditioning, TKO mice responded to the conditioned stimuli more strongly than WT mice in both the contextual learning test (day 2) and cued learning test (day 3) and showed higher freezing rates (Additional file 1: Fig. S3a–f). However, we could not conclude from this study that fear learning was enhanced in TKO mice because the freezing rate was high from the habituation period on day 1 (Additional file 1: Fig. S3a, b) and the period before cueing on day 3 (Additional file 1: Fig. S3e).

### Prepulse-inhibition (PPI) was impaired in TKO mice

**Fig. 6:**
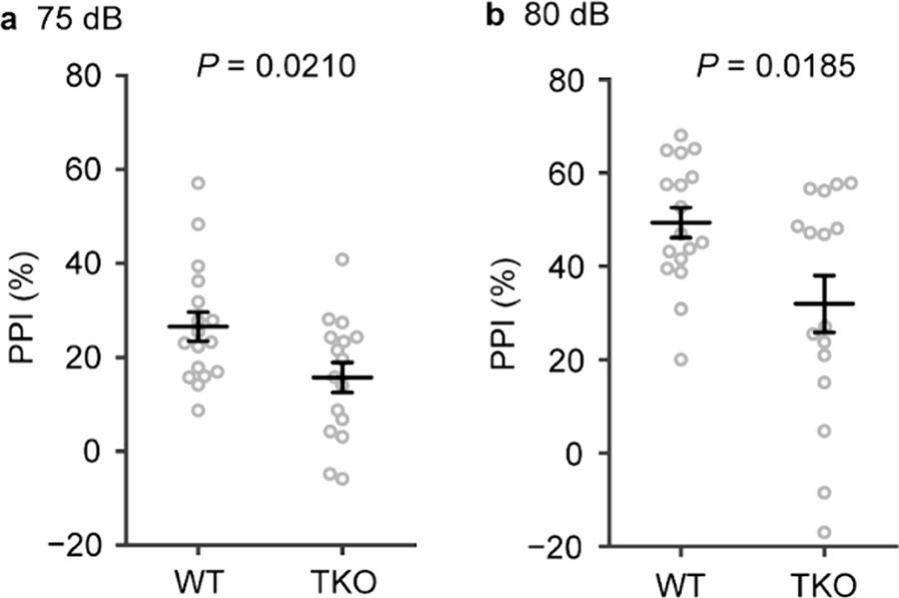
Prepulse inhibition (PPI) was impaired in TKO mice.** a**, **b** In the PPI test, TKO mice showed a lower inhibition rate compared to WT mice in the tests using 75-dB (**a**) and 80-dB (**b**) prepulses. This is a commonly observed endophenotype in patients with schizophrenia and mouse models of schizophrenia. All data are presented as the mean ± standard error of the means (WT mice, n = 17; TKO mice, n = 16). *P* values for differences between genotypes were determined by Welch’s *t*-test (**a**, **b**)

The PPI test is an experiment performed to measure the function of sensory/motor gating. The impairment of the gating is known to occur in several neuropsychiatric diseases, including schizophrenia [21]. In *Clstn* TKO mice, although the amplitude of the acoustic startle response was comparable to that of WT controls (Fig. 2d), the PPI of the acoustic startle response was markedly reduced (Fig. 6a, b).

### Nest-building behaviors were impaired in TKO mice

**Fig. 7:**
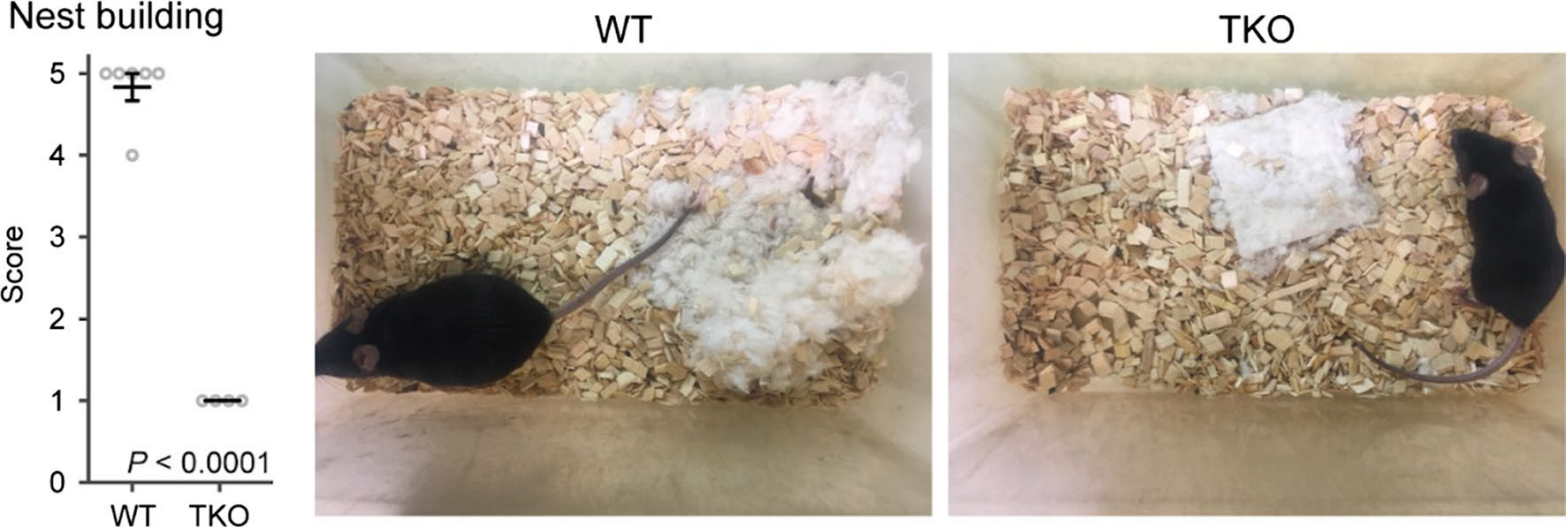
Nest-building behavior was impaired in TKO mice. Nest building was impaired in TKO mice. All data are presented as the mean ± standard error of the mean (WT mice, n = 8; TKO mice, n = 8). *P* values for differences between genotypes were determined using Welch’s *t*-test

Nest-building behavior is a social behavior, and the impairment in this behavior is observed in several model mice with mental disorders [22, 23]. TKO mice showed a significant defect in nest-building behavior (Fig. 7). That is, nestlets were left intact by all of the mutant mice, whereas all the WT mice formed nests within 24 h. These results indicate that social behavior is impaired in *Clstn* TKO mice.

### TKO mice did not show significant difference in the tail-suspension and Porsolt forced-swim tests

**Fig. 8:**
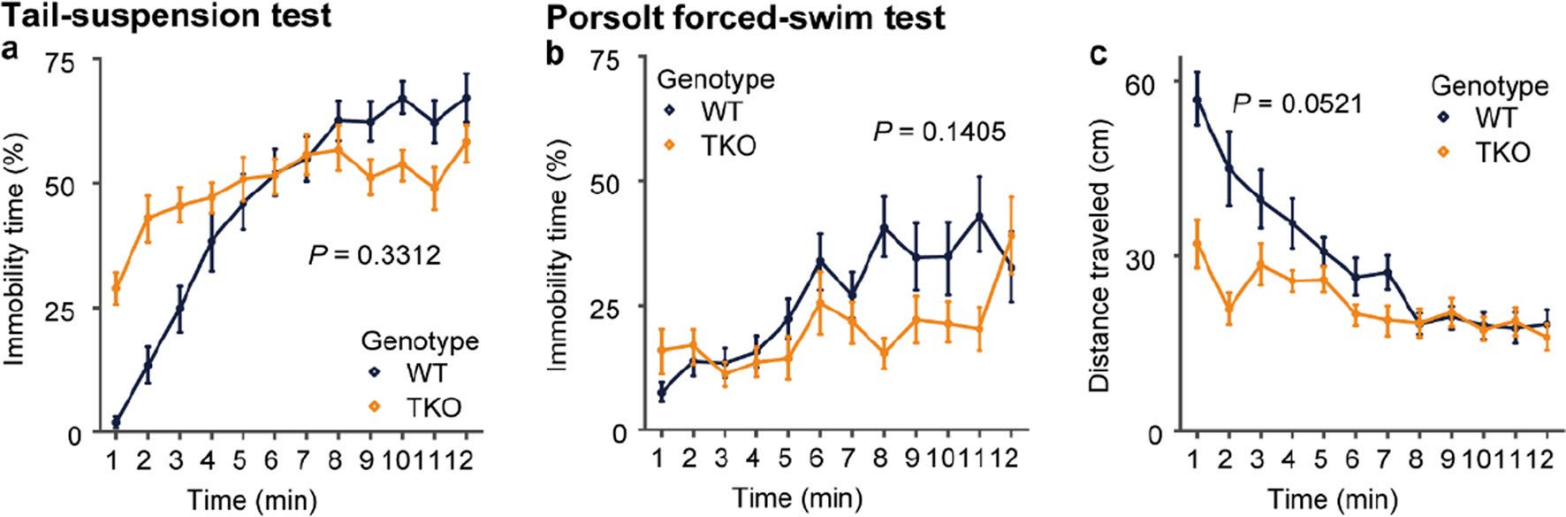
Depression-related behavioral test. Depression-like behavior in TKO mice. **a** Percentage immobility time (**a**) in the tail -suspension test. **b, c** Percentage immobility time (**b**) and distance traveled (**c**) in the Porsolt forced-swim test. All data are presented as the mean ± standard error of the mean (WT mice, n = 16 for **a** and n = 17 for **b**, **c**; TKO mice, n = 16). *P* values for differences between genotypes were determined by calculation of genotype effect in two-way repeated-measures analysis of variance (**a–c**)

In the tail-suspension and Porsolt forced-swim tests, which measure the depression-like state, TKO mice tended to differ from WT after the start of the test but did not show a significant difference overall (Fig. 8a–c).

### Altered number and distribution of cortical interneurons in *Clstn* TKO mice

To reveal the role of CLSTNs in neural development, we performed a series of histological experiments. First, we confirmed that TKO of the *Clstn*s did not affect the overall morphology of each brain region (Additional file 1: Fig. S4).

**Fig. 9:**
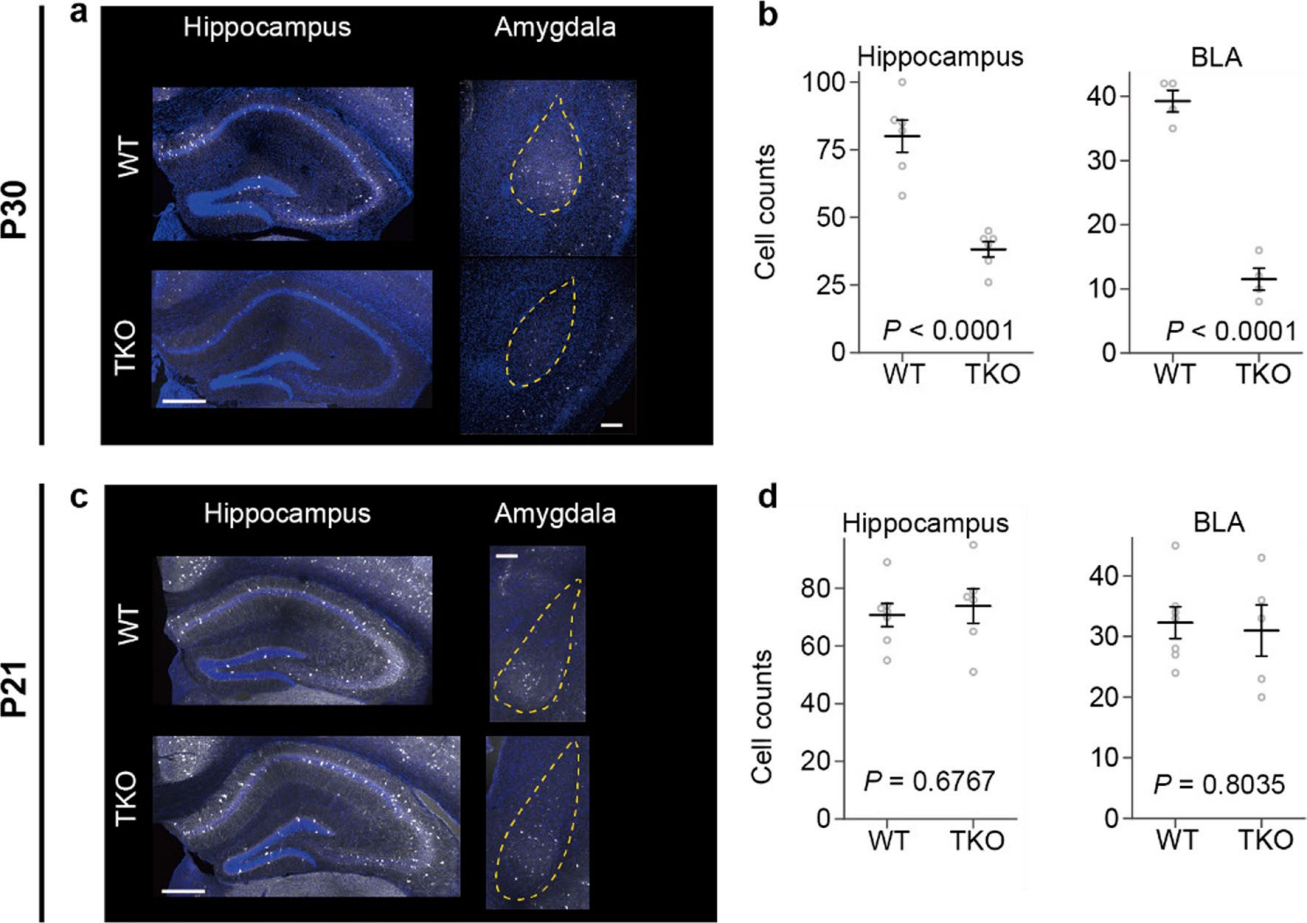
Reduced number of cortical interneurons in calsyntenin TKO mice.** a, b** Histological analysis of parvalbumin-positive (PV^+^) neuron numbers at P30. The number of PV^+^ neurons was reduced in the hippocampus and amygdala of TKO mice compared to that in WT mice. Scale bar 20 µm. **c, d** Histological analysis of PV^+^ neuron numbers at P21. The number of PV^+^ neurons in the TKO mice was comparable to that of PV^+^ neurons in WT mice. The area of the region of the interest was similar between WT and TKO mice at P21 and P30. All data are presented as the mean ± standard error of the mean [P21 (Hippocampus, WT: n = 7, TKO: n = 6; BLA, WT: n = 7, TKO: n = 6) and P30 (Hippocampus, WT: n = 6, TKO: n = 7; BLA, WT: n = 4, TKO: n = 4)]. *P* values for differences between genotypes were determined using Welch’s *t*-test (**b, d**)

Next, we counted the number of inhibitory neurons by immunostaining. We found that parvalbumin-positive (PV^+^) inhibitory neurons were decreased in *Clstn* TKO mice. The decrease was confirmed in the hippocampus and amygdala (Fig. 9a, b). The reduction of PV^+^ neurons and impaired PPI (Fig. 6) observed in TKO mice are typical endophenotypes of schizophrenia patients, as it is known that PV^+^ neurons are reduced in the postmortem brains of patients with schizophrenia [24, 25].

To reveal the cause of the loss of PV^+^ interneurons, we counted the number of PV^+^ interneurons in the early developmental stages. PV^+^ neurons are generated at embryonic day (E) 11 and migrate to their final destination by postnatal day (P) 14. We found that the number of PV^+^ neurons did not differ between WT and KO mice at P21 (Fig. 9c, d). Our results suggest that the loss of PV^+^ neurons in TKO mice occurs after the differentiation and migration of PV^+^ neurons. PV^+^ neurons are known to be vulnerable to stress, and this hypothesis is consistent with our behavioral observations that TKO mice are sensitive to stress (see below).

### *Clstn* TKO mice were hypersensitive to acute stress

**Fig. 10:**
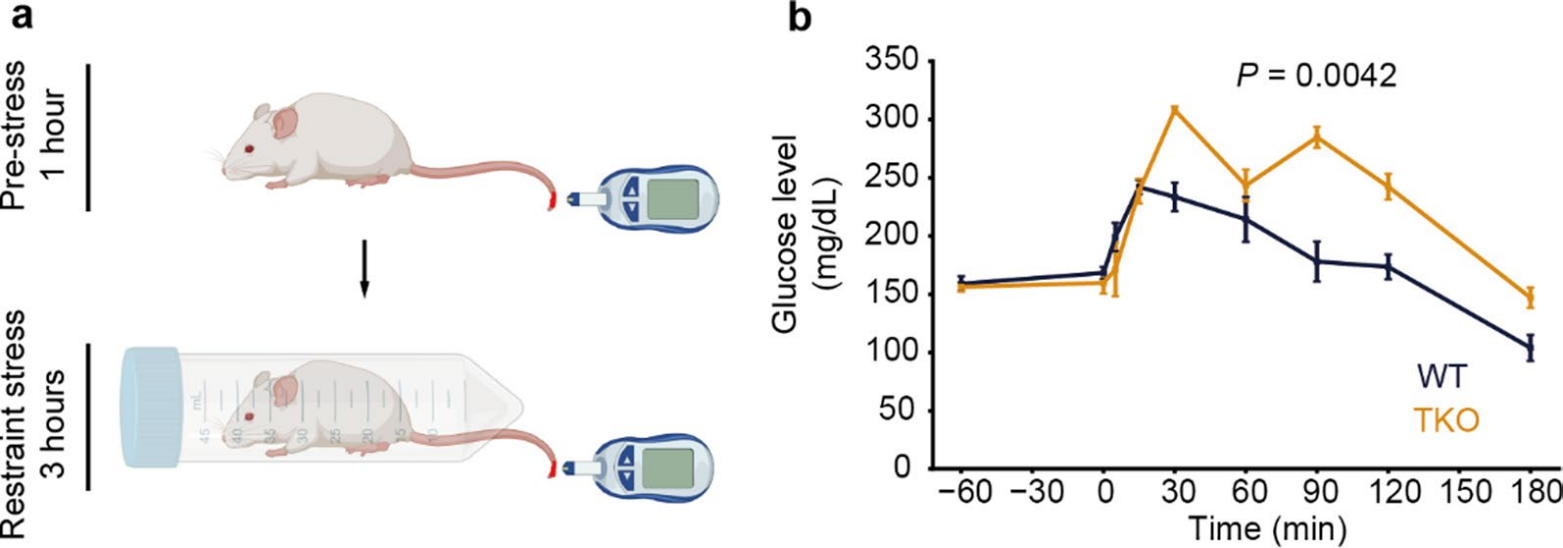
Acute stress response.** a** Schematic illustration of acute stress-response test. **b** In TKO mice, blood glucose levels significantly increased after acute stress, indicating hypersensitivity to stress. All data are presented as the mean ± standard error of the mean (WT mice, n = 3; TKO mice, n = 4). *P* values for differences between genotypes were determined by calculation of genotype effect in two-way repeated-measures analysis of variance

Several results described thus far suggest that the response to stress is altered in the TKO mice: early resignation of swimming in the water-maze test (Fig. 5), anxiety-like behavior (Fig. 3), and high immobility time in the beginning of the tail-suspension test (Fig. 8a). To test this hypothesis further, we investigated the acute stress response in TKO mice. Since stress increases the concentration of glucose in the blood via the hypothalamic–pituitary–adrenal (HPA) axis, the blood glucose concentration can be used as an indirect indicator of stress value. Furthermore, measuring blood glucose level is less invasive in mice and requires only a small amount of blood compared to measuring steroid hormone levels. Therefore, we assessed the stress response by measuring the increase in glucose concentration in the blood under acute stress (Fig. 10a). After exposure to acute stress, TKO mice showed abnormally higher blood glucose levels compared to WT mice, which indicates that the homeostasis system of TKO mice is hypersensitive to perturbation by external stress (Fig. 10b). TKO mice also showed a higher blood glucose level than WT mice, even under mild restraint stress, such as an injection of phosphate-buffered saline (PBS) during the insulin-tolerance test (Additional file 1: Fig. S5). These results suggest that TKO mice are more sensitive to stress than WT mice.

## Discussion

In the present study, we generated TKO mice by simultaneously knocking out all three *Clstn* paralogs and revealed several new phenotypes including low motivation during the learning task and hypersensitivity to acute stress. The hyperactivity and low-PPI phenotypes observed in the present study have already been reported for a single mutant of *Clstn2* [16], whereas low motivation and hypersensitivity to stress are newly found phenotypes. The involvement of CLSTNs in these phenotypes has not been reported in previous studies [6, 16, 20], likely due to the functional redundancy of CLSTNs.

### Novel phenotypes found in the TKO mice

The novel phenotypes found in our TKO mice were low motivation during the learning task and hypersensitivity to acute stress. These behavioral phenotypes observed in this study are also found in humans with neurological disorders, such as posttraumatic stress disorder and depression. Although the neural mechanisms underlying these disorders and the treatment for them have been widely studied, they remain unclear. Furthermore, although the association between motivation and response to stress has also been studied, the underlying mechanism(s) is still unclear [26, 27].

There are two major possible mechanisms underlying these behavioral abnormalities in TKO mice, which are not mutually exclusive. The first possible mechanism is that alterations in intracellular trafficking may lead to changes in neural responses, including stress responses. Previous studies have shown that CLSTNs play an important role in the intracellular trafficking of receptors such as GABA_B_Rs and NMDARs [13, 14]. CLSTN1 functions as an adapter protein in the axonal trafficking of APP [11–13], NMDARs [14], and GABA_B_Rs [13]. These functions of CLSTNs in intracellular trafficking have been reported for CLSTN1, but the detailed mechanism(s) and involvement of CLSTN2 and CLSTN3 have not been clarified. In addition, the intracellular trafficking of other unidentified molecules may be hampered by the loss of CLSTNs. It is known that CASY-1, an ortholog of CLSTN in *C. elegans*, interacts and transports insulin receptors in axons [17]. In the future, a rescue experiment for each CLSTN would identify CLSTN paralogs responsible for intracellular trafficking using a primary culture system from each brain region obtained from the TKO mice.

Second, neurodegeneration may cause behavioral abnormalities because CLSTNs are involved in cell stability. In this study, we found that the loss of PV^+^ neurons occurs after the differentiation and migration of PV^+^ neurons. Previous studies have also suggested that CLSTN2 and CLSTN3 are involved in cell stability through their functions in cell adhesion and synapse formation [4, 6, 28]. We will discuss the role of CLSTN2 in PV^+^ neuron stability in a later section.

As an extension of the current study, we consider it important to examine the functions of CLSTNs in the hypothalamus, hippocampus, amygdala, and prefrontal cortex. CLSTNs are expressed in the regions listed above in an overlapping manner [3]. Importantly, the paraventricular nucleus in the hypothalamus, a part of the HPA axis, is known to regulate the stress response. It is also known that the amygdala, especially the basolateral amygdala (BLA), plays an important role in both motivated behavior and stress responding [27]. Furthermore, GABAergic interneurons in the BLA are known to play an important role in controlling fear, anxiety, and motivation. Our study showed an altered number of PV^+^ GABAergic interneurons in the BLA of TKO mice, indicating that the excitatory/inhibitory balance is disrupted in the amygdala circuits. Furthermore, the hippocampus is also involved in the regulation of stress response; inhibition of the principal neurons of the dentate gyrus and CA3 region is required to suppress anxiety, and the inhibition of CA1 pyramidal neurons is required to suppress fear responses [29]. CLSTNs may be involved in the recapitulation of the behavior via intracellular trafficking or stability of PV^+^ interneurons. Taken together, the next step will be to determine which neural circuits and regions are affected by changes in the number of neurons.

### Phenotypes consistent with previous studies

In addition, the phenotypes observed in this mouse model, such as decreased PPI and loss of PV^+^ neurons (discussed later), are typical endophenotypes found in schizophrenic patients and *Clstn2*-deficient mice [16]. The biological mechanisms of schizophrenia and its treatment remain to be elucidated. In the future, clarifying the causes of the abnormality in our mouse model would shed light on these issues.

The impairment of the maintenance of PV^+^ neurons is most likely a cause of the schizophrenia-like endophenotype in TKO mice. In fact, the loss of PV^+^ neurons is one of the major histological phenotypes observed in the postmortem brains of patients with schizophrenia [24, 25] and autism spectrum disorder [30]. Furthermore, a recent report has shown morphological alterations of synaptic complexes in *Clstn2*-KO mice [28]. The study has reported that the loss of CLSTN2 impairs the morphology of synaptic complexes, including a reduction in the density of inhibitory synapses, length of synaptic contacts, and postsynaptic density [28]. These morphological alterations in inhibitory neurons may cause disturbances in the stability of inhibitory neurons. We also found that the loss of PV^+^ neurons occurs after the differentiation and migration of PV^+^ neurons, which supports the hypothesis that the loss of CLSTNs affects the stability of PV^+^ neurons. Several lines of work showed that PV^+^ neuron hypofunction in ASD and schizophrenia model mice. In mice, programmed cell death of PV^+^ neurons occurs around P7 to P14 [30]. In TKO mice, there might be disruption in this process. Also, in some ASD and schizophrenia model mice, expression levels of PV protein are lower than WT mice [30]. It should be noted that it is not elucidated whether the reduced number of PV neurons in TKO mice reflects degeneration of PV^+^ interneurons or merely a decrease in PV protein expression. The loss of PV^+^ neurons leads to disruption of the excitatory/inhibitory balance, resulting in major impairment of brain function [31]. Taken together, it is important to clarify the molecular machinery that maintains the stability of PV^+^ neurons, and it will be interesting to determine the function of CLSTNs in the cellular machinery.

### Limitation and future directions for elucidating the CLSTNs’ function

Several points could not be clarified by the battery of behavioral experiments in this study. We will discuss directions for future consideration of these points.

The Morris water maze test could not evaluate spatial memory because TKO mice gave up exploratory behavior early in the test. The Barnes maze test or eight-arm radial maze test is a spatial memory test that could be used for behavioral phenotyping of spatial memory for TKO mice.

A test used in Anisman and Merali [32] would be suited to approach further the low motivation observed during the Morris water maze. The test measures the amount of motivation to avoid an aversive environment. Also, they pharmacologically tested the involvement of dopamine and noradrenaline in this behavior. The low motivation phenotype observed for the first time in this study is very interesting, and pharmacological characterizations may reveal the neural circuits involved in this behavioral phenotype.

The next point is about the mechanisms underlying nest-building deficits in TKO mice. The result that nests building behavior completely disappeared was one of the striking points in this study. Sotelo et al*.* [33] recently showed that glutamatergic ensembles in the lateral hypothalamus are responsible for motivation of nest building. How these ensembles regulate complex nest-building behavior as a circuit is still unclear. It would be essential to investigate how CLSTN 1–3’s molecules are involved in this circuit as a future direction.

To elucidate the cause of behavioral disruption, we should discriminate between effects derived from the loss of inhibitory neurons and effects derived from the disruption of intracellular or synaptic machinery. The behavioral battery test was carried out with animals aged > 9 weeks. On the other hand, we found a decrease of inhibitory neurons at P30 but not at P21. Therefore, an interesting experiment would be to determine if there are behavioral disruptions that appear before inhibitory neuron loss occurs.

Finally, in this study, we could not elucidate the physiological function of the CLSTNs. The electrophysiological characterization (Additional file 1: Fig. S2c) suggested no difference in the excitatory neurotransmission mediated by AMPA receptors. On the other hand, inhibitory neurotransmission has not been addressed and should be evaluated by measuring mIPSCs in the future. Furthermore, in this study, functional dissection of each CLSTN paralog was not performed, but it would be meaningful to investigate the unique and redundant functions of each CLSTN. A functional dissection would be a good approach, such as partial rescue from the TKO. The TKO mice generated in this study will be a valuable resource for these experiments.

### Applicability of the model mice produced in this study

Our TKO mice can be used to shed light on the molecular function of CLSTNs in the CNS, as it is necessary to take into account the functional redundancy between paralogs to clarify the function of CLSTNs.

In addition, CLSTNs remain enigmatic molecules whose functions are yet to be fully elucidated. Recently, it was found that CLSTN plays an important role not only in the CNS but also in other organs, such as adipose tissue [19]. For example, Zeng et al*.* [19] have reported that CLSTN3β shows chaperone-like activity toward S100b, which acts as a neurotrophic factor to stimulate sympathetic axon growth and regulates sympathetic innervation in adipose tissue. CLSTNs, especially CLSTN1, are also partially expressed in other tissues outside the CNS [3]. These reports indicate that CLSTN will continue to be an interesting molecule not only in neuroscience but also in other fields. Furthermore, it is expected that simple analysis of single mutants in mammals will make it difficult to elucidate their functions due to functional redundancy among paralogs, and we believe that the TKO mice generated in this study that simultaneously lack all CLSTN paralogs will be useful for future studies.

## Materials and methods

### Animals and experimental design

All procedures involving animals were performed according to the methods approved by the Animal Care and Use Committee of the University of Tokyo. All behavioral tests were performed with mice that were 9–11 weeks old at the start of testing. Postnatal day 21 (P21) mice were used in immunohistological experiments. Mice were group-housed in a room with a 12-h light–dark cycle, with access to water and food ad libitum. The room temperature was maintained at 23 ± 1 °C.

### Generation of *calsyntenin* TKO mice

To reveal the function of CLSTNs, we generated a *Clstn1/Clstn2/Clstn3* TKO mouse line using the CRISPR/Cas9 system. To confirm the efficacy of single-guide (sg) RNAs for three *Clstn* genes in double-strand breaks at the target sites, green fluorescence reconstitution by homology-directed repair (HDR) of an enhanced green fluorescent protein (EGFP) expression cassette was employed [34]. The pX330 plasmid [35], which harbors the human codon-optimized *Streptococcus pyogenes* Cas9 cDNA (hCas9) and a targeting sgRNA, and the pCAG‐EGxxFP plasmid (a gift from Prof. Masahito Ikawa) containing an sgRNA target sequence between split EGFP fragments were cotransfected into HEK293T cells. EGFP fluorescence, which resulted from cutting and repair via HDR upon overlapping EGFP sequences in the split EGFP cassette, was observed after transfection. Three sgRNAs with the following sequences as targeting sequences were selected for zygote injection.

*Clstn1*: gaagtcgtacaaggcggccgtgg.

*Clstn2*: tgactgtggtgcggggcctcggg.

*Clstn3*: cagacgctccacaaagaccgggg.

C57BL/6NJcl (B6/N) embryos were injected with a mixture of RNAs: mRNA encoding *Cas9* (100 ng/µL), *Clstn1*-sgRNA (20 ng/µL), *Clstn2*-sgRNA (20 ng/µL), and *Clstn-3*-sgRNA (20 ng/µL). We obtained multiple KO mice and crossed them to generate *Clstn* TKO mice. Deletion was confirmed by polymerase chain reaction, DNA sequencing, and Western blotting.

The primers we used for genotyping and genome sequencing are listed below.

*Clstn1* forward primer: CAATCAGCTCATGTCCGGGT.

*Clstn1* reverse primer: CCCTGCACTGGATACTCAGC.

*Clstn2* forward primer: CCAAGCCCCTGATGGGTAAC.

*Clstn2* reverse primer: GCCGTGGGTGGACATACTTA.

*Clstn3* forward primer: CAGGCTTATGACTGTGGCGA.

*Clstn3* reverse primer: GAGGATCAAGTCAGTCATCGGG.

### Western blotting

Mouse brains were isolated and homogenized in lysis buffer containing 50 mM Tris–HCl (pH 7.5), 150 mM NaCl, 1% Triton X-100, protease inhibitor mixture (complete ethylenediaminetetraacetic acid-free, Roche), and phosphatase inhibitor cocktail mixture (PhosStop, Roche). The suspension was centrifuged at 12,000×*g* for 20 min at 4 °C. After denaturation at 95 °C for 5 min, the protein extracts were resolved by sodium dodecyl sulphate–polyacrylamide gel electrophoresis and transferred to a polyvinylidene fluoride membrane (Immobilon-P, Millipore). Blocking was performed by shaking in 5% skim milk/tris-buffered saline/Tween 20 (TBS-T) for 1 h at room temperature. The primary antibody reaction was then performed at 4 °C overnight. The secondary antibody reaction was performed at room temperature for 1 h. Information on the antibodies used in the antibody reactions is shown in Table 1. The following peptides were synthesized: 956–979 aa for Calsynthenin-1 (GenBank #OL981318), 934–954 aa for Calsynthenin-2 (GenBank # OL981319), and 939–956 aa for Calsynthenin-3 (GenBank # OL981320). Synthetic peptides coupled to keyhole limpet hemocyanin were used for antigens. Immunization and affinity purification were performed as reported previously [36]. All antibodies were diluted in 5% skim milk/TBS-T, and the dilution rates or concentrations were as follows: all antibodies against CLSTN1, CLSTN2, and CLSTN3 were adjusted to 1 μg/mL, and anti-β-actin antibodies were adjusted at a dilution of 1:5000. Anti-guinea pig IgG and anti-rabbit IgG antibodies were used at a dilution of 1:10,000 and anti-mouse IgG antibodies at a dilution of 1:20,000. Detection reactions were performed using the enhanced chemiluminescence (ECL) method (Amersham Hyperfilm ECL, GE Healthcare).

**Table 1:** Reagent and resources

Molecule	Host	Reagent or resource	Source	Identifier
CLSTN1	Guinea pig	Polyclonal guinea pig anti-CLSTN1	Prof. Masahiko Watanabe	
CLSTN2	Guinea pig	Polyclonal guinea pig anti-CLSTN2	Prof. Masahiko Watanabe	
CLSTN3	Rabbit	Polyclonal rabbit anti-CLSTN3	Prof. Masahiko Watanabe	
β-actin	Mouse	Monoclonal mouse anti-β-actin antibody	Sigma-Aldrich	A2228
Parvalbumin	Goat	Polyclonal goat anti-parvalbumin	Prof. Masahiko Watanabe	RRID: AB_2571614
Guinea Pig IgG (H + L)	Donkey	Peroxidase AffiniPure Donkey Anti-Guinea Pig IgG (H + L)	Jackson ImmunoResearch Laboratories, Inc	706-035-148
Mouse IgG (H + L)	Donkey	Peroxidase AffiniPure Donkey Anti-Mouse IgG (H + L)	Jackson ImmunoResearch Laboratories, Inc	715-035-151
Rabbit IgG (H + L)	Donkey	Peroxidase AffiniPure Donkey Anti-Rabbit IgG (H + L)	Jackson ImmunoResearch Laboratories, Inc	715-035-152
Goat IgG (H + L)	Donkey	Cy™5 AffiniPure Donkey Anti-Goat IgG (H + L)	Jackson ImmunoResearch Laboratories, Inc	705-175-147

### Electrophysiology

Electrophysiological experiments were performed using 8–10-week-old animals, following previously described methods [37]. Briefly, hippocampal slices were prepared in Krebs–Ringer solution saturated with 95% oxygen (O_2_) and 5% carbon dioxide (CO_2_) using a tissue slicer (Leica VT1200S; Leica Biosystems, Nussloch, Germany). After a 1-h recovery period at 25 °C, the slices were transferred to a recording chamber filled with Krebs–Ringer solution (which contained 119 mM NaCl, 2.5 mM KCl, 2.5 mM CaCl_2_, 1.3 mM MgSO_4_, 26.2 mM NaHCO_3_, 1 mM NaH_2_PO_4_, and 11 mM glucose) saturated with 95% O_2_ and 5% CO_2_. The same solution was used for recordings. To prevent bursting activity, the CA3 region was surgically cut off. Synaptic responses were recorded in the stratum radiatum of the CA1 region using the extracellular field-potential recording technique. The glass recording pipette was filled with 3 M NaCl. For LTP measurements, 100 μM picrotoxin (Sigma-Aldrich) was added to the extracellular solution to block GABA_A_ receptor-mediated inhibitory synaptic responses. To evoke a synaptic response, Schaffer collateral-commissural fibers were stimulated at 0.1 Hz (test pulse) with a bipolar tungsten electrode, and the slope value of EPSPs was adjusted between 0.10 and 0.15 mV/ms. After establishing a stable baseline, tetanic stimulation (100 Hz for 1 s) was applied to induce LTP. Synaptic responses were recorded using Axopatch-1D amplifiers (Molecular Devices, Sunnyvale, CA, USA), and the signal was digitized at 10 kHz with Digidata 1440A (Molecular Devices) and analyzed using pClamp10 (Molecular Devices).

### Immunostaining and image analysis

Under deep anesthesia with sodium pentobarbital (100 mg/kg body weight, i.p.), animals were perfused with 4% paraformaldehyde dissolved in 0.1 M phosphate buffer (pH 7.4), and brain tissue was post-fixed for 24 h. Fixed brain blocks were then cryoprotected in 30% sucrose and sectioned at 30 µm thickness using a cryostat.

Sections were permeabilized by floating sections in 0.2% Triton-X100 in PBS and shaken at room temperature for 15 min. The primary antibody reaction was performed at 4 °C overnight or overnight with shaking, and the secondary antibody reaction was performed by shaking at room temperature for 1 h. Information on the antibodies used in the antibody reactions is shown in Table 1. All antibody dilutions were performed in 5% bovine serum albumin and 0.2% Triton-X100 in PBS. Anti-parvalbumin antibody was used at 1 μg/mL. All secondary antibodies were diluted at a dilution factor of 1:1,000; Hoechst33258 (Sigma-Aldrich, 861405) was diluted to a dilution factor of 1:2000. Sections were counterstained with Hoechst and examined using a confocal laser scanning microscope (SP5: Leica).

All image analyses were performed with the experimenter blinded to the genotype. The cell numbers were quantified for each brain region. The analysis was performed using ImageJ and Python software. All data are reported as the mean ± standard error of the mean (SEM).

### Behavioral assays

All behavioral assays were performed with male mice aged > 9 weeks and under blind conditions, where the experimenter did not recognize the mouse genotype. All behavioral tests were performed between 10 am and 7 pm. All subjects were mixed at 3 weeks and housed three to four per cage. 33 mice were used in behavioral battery tests.

#### Wire-hang test

A wire-hang apparatus (O’Hara & Co., Tokyo, Japan) was used to measure arm muscular strength. Mice were placed on a 10 × 10 cm square grid ceiling (30-cm height), which was then inverted, and the time to fall was measured with a 60 s cutoff time. Each mouse was measured twice, and the average was taken as the score.

#### Open-field test

Each animal was placed in a 25-cm square automated measuring device (O’Hara & Co., Tokyo, Japan), and its behavior was captured by a camera mounted on the top of the device for 15 min. The brightness of the floor was set to 200 lx. The movement distance, speed, and time spent in the central area were calculated based on the center coordinates of the mouse.

#### Elevated plus-maze test

Each mouse was placed in the center of a cross-armed maze apparatus (O’Hara & Co., Tokyo, Japan) consisting of two open-arms (5-cm wide, 25-cm long, and 0-cm high on the wall), two closed-arms (5-cm wide, 25-cm long, and 15-cm high on the wall), and a center square (5 × 5 cm) that was 25 cm above the ground, and activity was recorded for 15 min using a camera mounted on top. Arms of the same type were arranged on the opposite sides of the apparatus. The total distance traveled, time spent in the open arms, and time spent in the closed arms were calculated from the center of the subject.

#### Light–dark preference test

Each mouse was placed in an automatic measuring device (O’Hara & Co., Tokyo, Japan), which had a partition in the center of the long side and separates a dark room and a light room (20 × 20 cm square in each room), and its movement was recorded for 15 min using a camera installed in the upper part of the device. The total distance traveled, time spent in the light chamber, and time spent in the dark chamber were calculated from the trajectory of the subject.

#### Rotarod test

Each mouse was placed on a rotating rod (Accelerating Rotarod; Basile, Varese, Italy), which has an 8.5-cm wide bar between two 18-cm high walls, and the latency to fall off the rod during the acceleration from 4 to 40 rpm over 5 min was measured in four trials per day at an interval of 2 h for 2 days (8 trials in total).

#### Morris water-maze test

The water-maze test was conducted using a 100-cm diameter pool and a 10-cm diameter platform. Water was filled to a depth of 1 cm on the platform. The water temperature was set to 20 °C, and the platform was hidden by making the water cloudy with nontoxic white paint. We also placed several markers around the maze. Each mouse was placed in the pool, and its movement was video-recorded with a camera placed at the top of the pool. The position of the platform varied between mice, but was constant for each mouse. When the mice did not get on the platform within the trial time, they were forced to move to the platform for 30 s and returned to their home cage. After the final trial on the sixth day, we conducted a probe test. In the probe test, the platform was removed and the mouse explored for the platform for 1 min, and the movement of the mouse was video-recorded from the top of the platform. For analysis, the time to reach the platform, trajectory, and immobility time were calculated from the center of the mouse.

#### Acoustic startle and prepulse-inhibition (PPI) test

The startle response and prepulse-inhibition tests were performed using the Startle Monitor System (Hamilton-Kinder, LLC, Poway, CA, USA 100). Mice were kept in a chamber (27-cm wide, 27-cm high, 16-cm deep) and stimulated by the sound from the speaker placed 15 cm above the chamber. The force (N) at which the floor was pushed by the startle response of the mice was recorded, and the maximum value of each trial was considered as the startle response. Each session was followed by an adaptation period of 5 min and a trial period consisting of 56 trials. A 70-dB white noise was used as the background noise in the chamber. The following three trial patterns were used. (1) No stimulus trial, (2) startle stimulus (90, 100, 110, or 120 dB) alone, and (3) prepulse (75 dB or 80 dB) + startle stimulus (120 dB) trial. The number of times for each type of trial was as follows, and the order of the trials was given in random order (1) four trials without stimulus, (2) four trials with stimulus of each intensity, and (3) four trials with each prepulse + stimulus of each intensity. The interval between each trial was set at 10–20 s. The percent PPI was calculated according to the following formula: PPI (%) = 100 × (1 – [{startle response amplitude in prepulse + startle trial}/{startle response amplitude in startle stimulus–only trial}]).

#### Contextual and auditory fear-conditioning test

Fear association learning tests were conducted using an automatic measuring device (O’Hara & Co., Tokyo, Japan) over a period of 3 days. A chamber (10-cm in width, 10-cm in height, and 10-cm in depth) was placed in the apparatus, and a mouse was placed in the chamber. Two types of chambers were prepared as follows: chamber A was a translucent chamber, with a stainless-steel grid floor (bars 0.2 cm in diameter, spaced 0.5 cm apart) for electrical stimulation, and chamber B was an opaque chamber with a bedding underneath. During the experiment, the behavior of the subject was recorded using a camera at the top of the measuring apparatus. The conditioned stimulus (CS) was provided by a speaker at the top of the instrument (65 dB). The electrical stimulus (35 mA) was administered during the last 2 s of the CS and served as the unconditioned stimulus (US). In each test, the percentage of freezing time and distance traveled were calculated automatically.

Day 1: The fear conditioning.

Each mouse was placed in chamber A and then video-recorded in the measuring apparatus for 4 min. CS and US (electrical stimuli) were administered 3 min after the start of the test.

Day 2 Context-fear association test.

Each mouse was placed in the same chamber A as used on the previous day (same context) and the behavior was observed for 6 min.

Day 3 CS-US association test.

To eliminate the influence of contextual memory, we changed the surrounding environment (light source position in the measuring apparatus, ventilation fan speed, chamber, and wall color) from the setting of day 1 (chamber B). We placed each mouse in chamber B and observed its behavior for 6 min. Three minutes later, the same CS (acoustic stimuli) as on day 1 was delivered to the mice.

#### Hot-plate test

Each mouse was placed in a cylindrical enclosure on a hot plate at 50 °C, and the latency to the first hind leg lick and jump behavior were measured.

#### Tail-flick test

The time from the start of the beam irradiation to the tail until the reflection occurred was measured using an automatic measuring device (Cat. No.7360: Ugo Basile, Comerio, Italy). Measurements were repeated three times, and the average of the measurements was used as the individual score.

#### Tail-suspension test

The measurements were performed using an automatic measuring chamber (O’Hara & Co.). Each mouse was suspended with its tail fixed in the chamber, and its immobility time was measured over a 10-min test period.

#### Porsolt forced-swim test

A cylinder (20 × 10 cm) filled with water at 20 °C up to a height of 7.5 cm was placed in an auto measuring chamber (O’Hara & Co.). Each mouse was placed in water, and the immobility time was measured over a 10-min test period.

#### Nest-building test

The cage in which the mouse was being housed was covered with 50 g of woodchip bedding and filled with Nestlet (Nesting Material, Ancare). Twenty-four hours later, the nest was evaluated in five levels using Deacon 2006 [38] as a reference.

#### Stress-response test

Mice were placed in 50-mL tubes and subjected to restraint stress. Blood was drawn from the tail vein over time, and blood glucose levels were measured using an Accu-Chek^®^ Aviva Nano. One hour before stressing the mice, the tail was cut, and blood sampling was started. The animals were not fed for 1 h prior to the stress exposure to avoid the effects of elevated blood glucose levels caused by food.

#### Insulin-tolerance test

Blood was drawn from the tail vein over time, and blood glucose levels were measured using a glucometer (Accu-Chek^®^ Aviva Nano). The mice were fasted for 1 h with water provided ad libitum. After measuring basal glucose levels, insulin (0.9 U/kg) was administered intraperitoneally, and glucose levels were measured 15, 30, 60, 90, and 120 min after the injection.

### Statistical data analyses

Statistical analyses were performed using a statistical package (Python, R, and Prism). Two-tailed t-tests, Dunnett’s tests, and repeated measures two-way analysis of variance with Greenhouse–Geisser correction were used. Error bars indicate the SEM. The detailed statistics are described in Additional file 1: Table S1.

## Supplementary Information

**Additional file 1: Fig. S1.** Both female and male TKO mice showed lower weight compared to wild-type mice. **Fig. S2**. Synaptic plasticity was normal in TKO mice. **Fig. S3**. TKO mice showed high freezing rate in the fear conditioning test. **Fig. S4**. Overall brain morphology was normal in TKO mice. **Fig. S5**. Insulin-tolerance test. **Table S1**. Statistical analysis related to Figures 2–10 and S1–2.

## Data Availability

All data in this study are available upon reasonable request.

## Abbreviations

BLA: Basolateral amygdala
CNS: Central nervous system
CS: Conditioned stimulus
E: Embryonic day
hCas9: Human codon-optimized *Streptococcus pyogenes* Cas9
HDR: Homology-directed repair
HPA: Hypothalamic–pituitary–adrenal
KO: Knockout
LNS: Laminin-α, neurexin, and sex hormone-binding globulin
LTP: Long-term potentiation
P: Postnatal day
PPI: Prepulse inhibition
PTSD: Posttraumatic stress disorder
PV^+^: Parvalbumin positive
SEM: Standard error of the mean
sgRNA: Single guide RNA
TKO: Triple knockout
US: Unconditioned stimulus
