# Supplementary material for: Loss of calsyntenin paralogs disrupts interneuron stability and mouse behavior
Source: Mol Brain. 2022 Mar 12;15:23. doi: 10.1186/s13041-022-00909-8 (PMC8917637; doi:10.1186/s13041-022-00909-8)
Supplement: Supplementary file 1 — Additional file 1: Fig. S1. Both female and male TKO mice showed lower weight compared to wild-type mice. Fig. S2. Synaptic plasticity was normal in TKO mice. Fig. S3. TKO mice showed high freezing rate in the fear conditioning test. Fig. S4. Overall brain morphology was normal in TKO mice. Fig. S5. Insulin-tolerance test. Table S1. Statistical analysis related to Figures 2–10 and S1–2. [file 13041_2022_909_MOESM1_ESM.docx]

**Loss of calsyntenin paralogs disrupts interneuron stability and mouse behavior**

**Additional file 1: Figures S1-5 and Table S1**


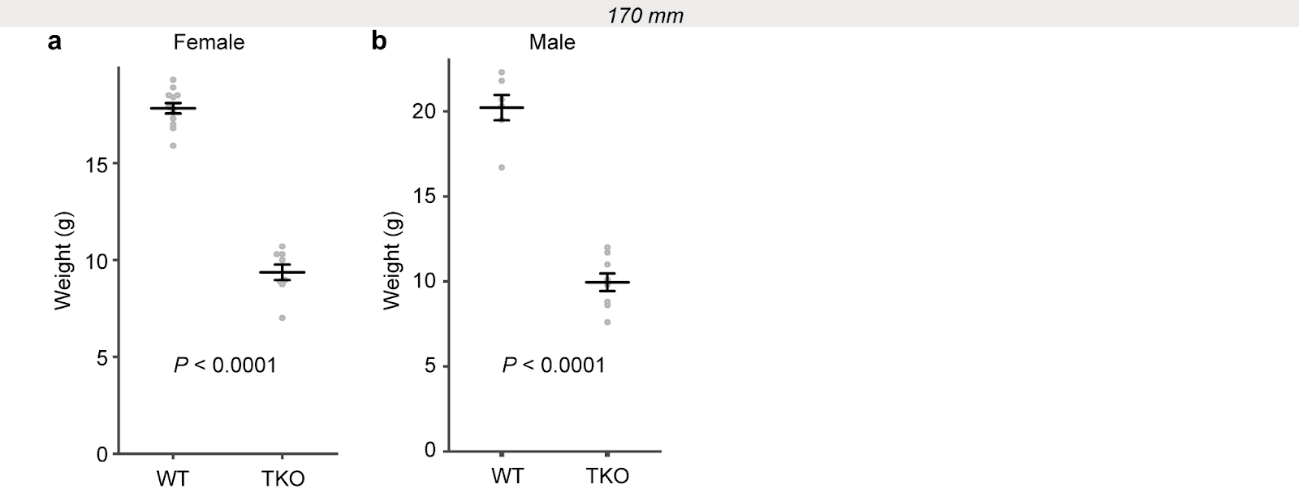


**Figure S1. Both female and male TKO mice showed lower weight compared to wild-type mice. a, b** Weights of the mice at P30. All data are presented as the mean ± standard error of the mean (**a**, WT mice, n =12; TKO mice, n =8; **b**, WT mice, n =6; TKO mice, n =8). *P* values for differences between genotypes were determined by Welch’s *t-*test.


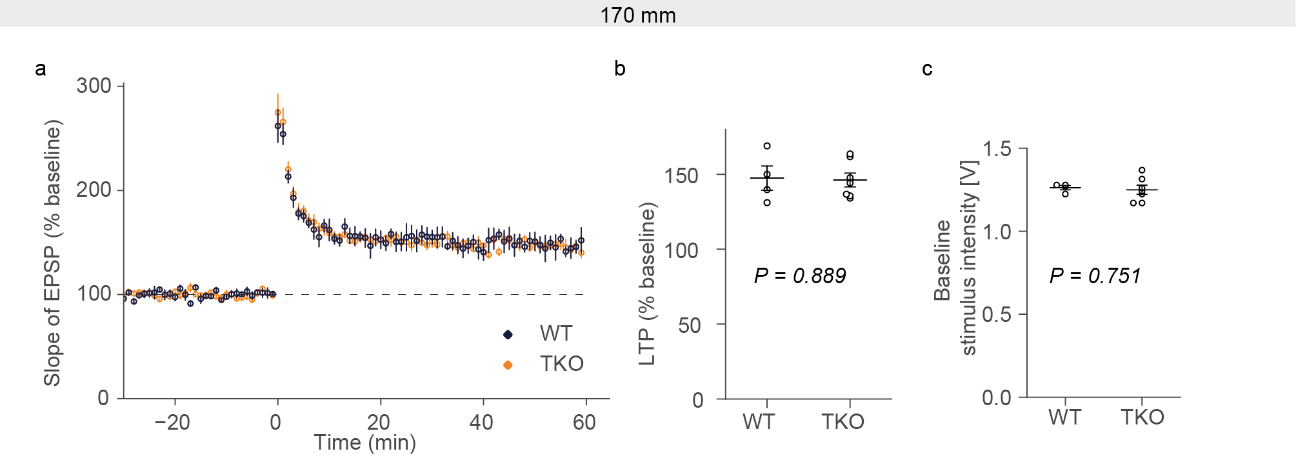
**Figure S2. Synaptic plasticity was normal in TKO mice.** **a** Time course of long-term potentiation (LTP) induced by tetanic stimulation. Tetanic stimulation was applied at time 0. **b** The ratio of LTP at 60 min after tetanic stimulation. The LTP ratio of TKO mice was comparable to that of WT mice. **c** The intensity of baseline stimuli applied to evoke EPSPs, whose slopes were indistinguishable between genotypes. The same level in WT and TKO mice suggests that there was no difference in AMPA receptor-mediated synaptic responses. All data are presented as the mean ± standard error of the mean (WT mice, n = 4; TKO mice, n = 7). *P* values for differences between genotypes were determined by Welch’s *t-*test.

**
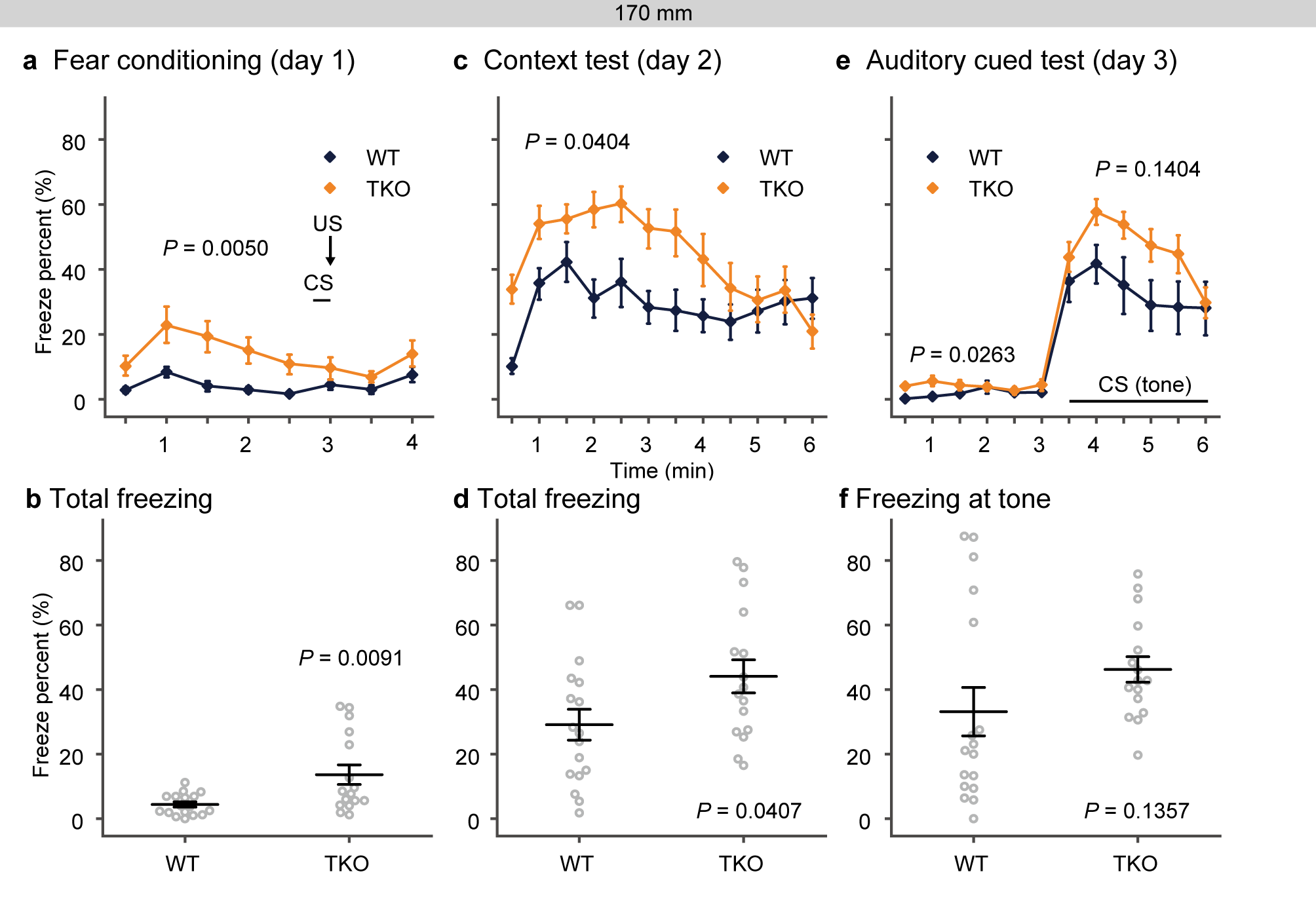
**

**Figure S3. TKO mice showed high freezing rate in the fear conditioning test. a**, **b** TKO mice showed a high freezing rate in a novel chamber environment. After fear conditioning, the freezing rate was similar to that of WT mice. **c, d** In a context-dependent fear-conditioning test, TKO mice showed a higher freezing rate than WT mice. **e, f** In a context-independent auditory cue fear-conditioning test, TKO mice also showed a higher freezing rate than WT mice. All data are presented as the mean ± standard error of the mean (WT mice, n = 17; TKO mice, n = 16). *P* values for differences between genotypes were determined by Welch’s *t-*test (**b**, **d**, **f**) or two-way repeated-measures analysis of variance (**a**, **c**, **e**).


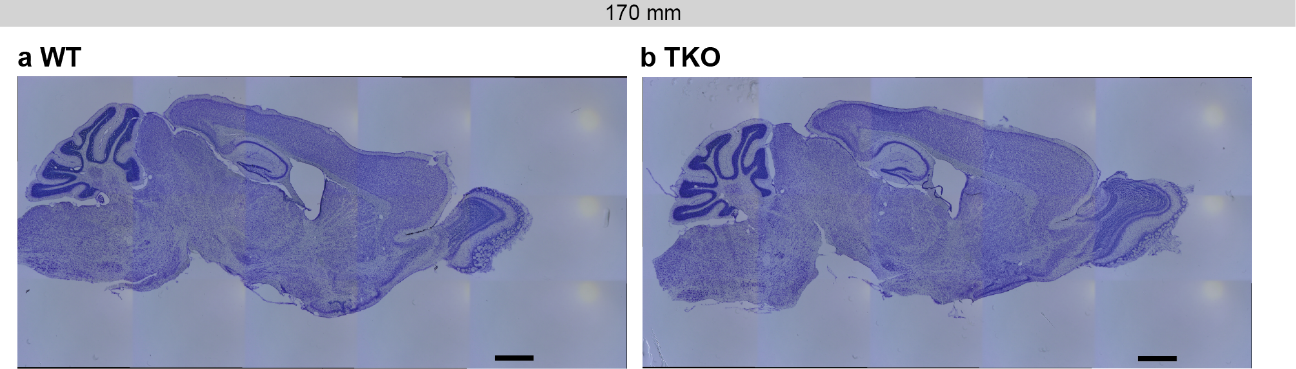


**Figure S4. Overall brain morphology was normal in TKO mice. a, b** Results of Nissl staining. No significant morphological differences were observed between the WT and TKO mouse brains. Scale bars, 1 mm.


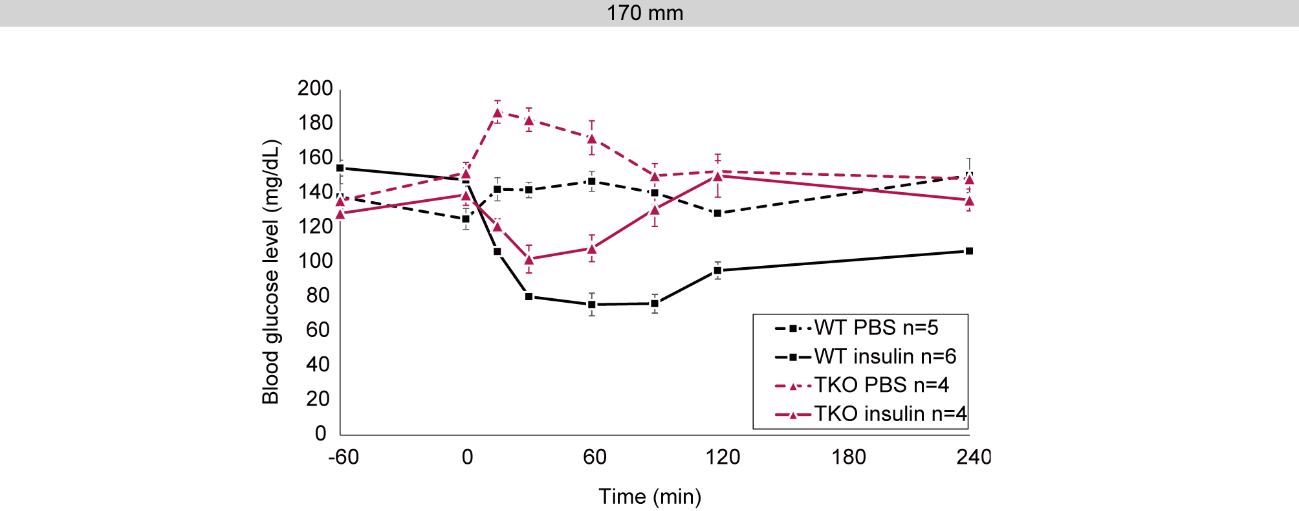


**Figure S5. Insulin-tolerance test**Result of insulin-tolerance test. TKO mice also showed a higher blood glucose level than WT mice, even under mild restraint stress, such as an injection of PBS during the insulin-tolerance test.

**Table S1. Statistical analysis related to Figures 2–10 and S1-2.**

| **Figure 2a** | Two-tailed Welch's *t*-test. *p <* 0.0001 WT: n=17, TKO: n=16. |
| --- | --- |
| **Figure 2b** | Wilcoxon rank-sum test. *p=* 0.0002  WT: n=17, TKO: n=16. |
| **Figure 2c** | Two-way repeated measures ANOVA.  Genotype: F_1,31_ = 5.555, *p=* 0.0249;  Trial: F_4.980,154,4_ = 63.61, *p <* 0.0001;  Genotype × Time interaction: F_7,217_ = 7.217, *p=* 0.8357;  WT: n=17, TKO: n=16. |
| **Figure 2d** | Two-way repeated measures ANOVA.  Genotype: F_1,31_ = 0.1832, *p*= 0.6716;  dB: F_4,124_ = 40.30, *p <* 0.0001;  Genotype × Time interaction: F_4,124_ = 0.3895, *p* = 0.8158;  WT: n=17, TKO: n=16. |
| **Figure 2e** | Two-tailed Welch's *t*-test. *p=*0.2941;  WT: n=17, TKO: n=16. |
| **Figure 2f** | Two-tailed Welch's *t*-test. *p=*0.1455;  WT: n=17, TKO: n=16. |
| **Figure 2g** | Two-tailed Welch's *t*-test. *p=*0.0013;  WT: n=17, TKO: n=16. |
| **Figure 3a** | Two-way repeated measures ANOVA.  Genotype: F_1,31_ = 3.849, *p=* 0.0588;  Time: F_5.031, 156.0_= 3.828, *p=* 0.0026;  Genotype × Time interaction: F_14,434_ = 4.082, *p <*0.0001.  WT: n=17, TKO: n=16. |
| **Figure 3b** | Two-tailed Welch's *t*-test. *p=* 0.0623;  WT: n=17, TKO: n=16. |
| **Figure 3c** | Two-tailed Welch's *t*-test. *p=*0.0208;  WT: n=17, TKO: n=16. |
| **Figure 3d** | Two-tailed Welch's *t*-test. *p=*0.0079;  WT: n=17, TKO: n=16. |
| **Figure 3e** | Two-way repeated measures ANOVA.  Genotype: F_1,31_ = 9.763, *p=* 0.0038;  Time: F_7.411, 229.8_ = 2.233, *p=*0.0302;  Genotype × Time interaction: F_14,448_ = 1.198, *p=*0.2730;  WT: n=17, TKO: n=16. |
| **Figure 3f** | Two-tailed Welch's *t*-test. *p=*0.0035;  WT: n=17, TKO: n=16. |
| **Figure 3g** | Two-tailed Welch's *t*-test. *p=* 0.1176;  WT: n=17, TKO: n=16. |
| **Figure 3h** | Two-tailed Welch's *t*-test. *p=*0.0063;  WT: n=17, TKO: n=16. |
| **Figure 3i** | Two-way repeated measures ANOVA.  Genotype: F_1,31_ = 3.572, *p=* 0.7569;  Time: F_7.411, 229.8_ = 13.69, *p <* 0.0001;  Genotype × Time interaction: F_14,434_ = 3.573, *p <* 0.0001;  WT: n=17, TKO: n=16. |
| **Figure 3j** | Two-tailed Welch's *t*-test. *p=*0.7572;  WT: n=17, TKO: n=16. |
| **Figure 3k** | Two-tailed Welch's *t*-test. *p=*0.0037;  WT: n=17, TKO: n=16. |
| **Figure 3l** | Two-tailed Welch's *t*-test. *p=*0.2030;  WT: n=17, TKO: n=16. |
| **Figure 4a** | Two-way repeated measures ANOVA.  Genotype: F_1,31_ = 14.28, *p=* 0.0007;  Time: F_5.434, 168.4_ = 10.46, *p <* 0.0001;  Genotype × Time interaction: F_14,434_ = 8.300, *p <* 0.0001;  WT: n=17, TKO: n=16. |
| **Figure 4b** | Two-way repeated measures ANOVA.  Genotype: F_1,31_ = 0.04062, *p=* 0.8416;  Time: F_8.316, 257.8_ = 1.823, *p <* 0.0702;  Genotype × Time interaction: F_14,434_ = 0.9431, *p=* 0.5120;  WT: n=17, TKO: n=16. |
| **Figure 4c** | Two-tailed Welch's *t*-test. *p=*08431;  WT: n=17, TKO: n=16. |
| **Figure 5a** | Two-way repeated measures ANOVA.  Genotype: F_1,31_ = 10.63, *p=* 0.0027;  Time: F_5.872, 182.0_ = 3.612, *p=* 0.0022;  Genotype × Time interaction: F_23,_ _713_ = 2.913, *p <* 0.0001;  WT: n=17, TKO: n=16. |
| **Figure 5b** | Two-tailed Welch's *t*-test. *p=*0.0003;  WT: n=17, TKO: n=16. |
| **Figure 5c** | Two-way repeated measures ANOVA.  Genotype: F_1,31_ = 21.86, *p <* 0.0001;  Time: F_12.08, 374.3_ = 5.104, *p <* 0.0001;  Genotype × Time interaction: F_23,_ _713_ = 3.004, *p <* 0.0001;  WT: n=17, TKO: n=16. |
| **Figure 5d** | Two-tailed Welch's *t*-test. *p <* 0.0001;  WT: n=17, TKO: n=16. |
| **Figure 6a** | Two-tailed Welch's *t*-test. *p=*0.0210;  WT: n=17, TKO: n=16. |
| **Figure 6b** | Two-tailed Welch's *t*-test. *p=*0.0185;  WT: n=17, TKO: n=16. |
| **Figure 7** | Two-tailed Welch's *t*-test. *p <*0.0001;  WT: n=8, TKO: n=8. |
| **Figure 8a** | Two-way repeated measures ANOVA.  Genotype: F_1,30_ = 0.9757, *p=* 0.3312;  Time: F_7.311, 219.3_ = 29.88, *p <* 0.0001;  Genotype × Time interaction: F_11,330_ = 8.208, *p <* 0.0001;  WT: n=16, TKO: n=16. |
| **Figure 8b** | Two-way repeated measures ANOVA.  Genotype: F_1,31_ = 2.288, *p=* 0.1405;  Time: F_8.240, 255.4_ = 7.826, *p <* 0.0001;  Genotype × Time interaction: F_19,589_ = 1.536, *p=* 0.0678;  WT: n=17, TKO: n=16. |
| **Figure 8c** | Two-way repeated measures ANOVA.  Genotype: F_1,31_ = 4.079, *p=* 0.0521;  Time: F_6.311,195.6_ = 18.53, *p <* 0.0001;  Genotype × Time interaction: F_19,589_ = 4.549, *p <* 0.0001;  WT: n=17, TKO: n=16. |
| **Figure 9b left** | Two-tailed Welch's *t*-test. *p <* 0.0001;  WT: n=6, TKO: n=7. |
| **Figure 9b right** | Two-tailed Welch's *t*-test. *p <* 0.0001;  WT: n=4, TKO: n=4. |
| **Figure 9d left** | Two-tailed Welch's *t*-test. *p=* 0.6767;  WT: n=7, TKO: n=6. |
| **Figure 9d right** | Two-tailed Welch's *t*-test. *p=*0.8035;  WT: n=7, TKO: n=6. |
| **Figure 10b** | Two-way repeated measures ANOVA.  Genotype: F_1,5_ = 24.8, *p=* 0.0042;  Time: F_5, 25_ = 8.958, *p <* 0.0001;  Genotype × Time interaction: F_5, 25_ = 2.249, *p=* 0.0807;  WT: n=4, TKO: n=3. |
| **Figure S1a** | Two-tailed Welch's *t*-test. *p <* 0.0001;  WT: n=12, TKO: n=8. |
| **Figure S1b** | Two-tailed Welch's *t*-test. *p <* 0.0001;  WT: n=6, TKO: n=8. |
| **Figure S2b** | Two-tailed Welch's *t*-test. *p=*0.889;  WT: n=4, TKO: n=7. |
| **Figure S2c** | Two-tailed Welch's *t*-test. *p=*0.751;  WT: n=4, TKO: n=7. |
| **Figure S3a** | Two-way repeated measures ANOVA.  Genotype: F_1,31_ = 9.123, *p=* 0.0050;  Time: F_3.987, 123.6_ = 5.609, *p=*0.0004;  Genotype × Time interaction: F_7, 217_ = 2.051, *p=*0.0501;  WT: n=17, TKO: n=16. |
| **Figure S3b** | Two-tailed Welch's *t*-test. *p=*0.0091;  WT: n=17, TKO: n=16. |
| **Figure S3c** | Two-way repeated measures ANOVA.  Genotype: F_1,31_ = 4.574, *p=* 0.0404;  Time: F_6.806, 211.0_ = 11.28, *p <*0.0001;  Genotype × Time interaction: F_11, 341_ =4.412, *p <*0.0001;  WT: n=17, TKO: n=16. |
| **Figure S3d** | Two-tailed Welch's *t*-test. *p=*0.0407;  WT: n=17, TKO: n=16. |
| **Figure S3e left** | Two-way repeated measures ANOVA.  Genotype: F_1,31_ = 5.442, *p=* 0.0263;  Time: F _3.992, 123.8_ = 0.5716, *p=*0.6834;  Genotype × Time interaction: F _5, 155_ = 1.159, *p=*0.3319;  WT: n=17, TKO: n=16. |
| **Figure S3e right** | Two-way repeated measures ANOVA.  Genotype: F_1,31_ = 2.289, *p=* 0.1404;  Time: F _3.228, 100.1_ =8.920, *p <*0.0001;  Genotype × Time interaction: F _5, 155_ = 2.144, *p=*0.0631;  WT: n=17, TKO: n=16. |
| **Figure S3f** | Two-tailed Welch's *t*-test. *p=*0.13577;  WT: n=17, TKO: n=16. |
